# Clinical and Physiological Predictors of Acetylcholine‐Induced Coronary Spasm in ANOCA/INOCA Patients: A Retrospective Cohort Study

**DOI:** 10.1002/ccd.70628

**Published:** 2026-04-12

**Authors:** Jeremie Buri, Aurelia Zimmerli, Thabo Mahendiran, Adil Salihu, Victor Weerts, Olivier Muller, David Meier, Stephane Fournier

**Affiliations:** ^1^ Department of Cardiology Lausanne University Hospital (CHUV) Lausanne Switzerland

**Keywords:** acetylcholine, ANOCA, coronary microvascular dysfunction, coronary vasospasm, INOCA

## Abstract

**Background:**

A substantial proportion of patients with angina and/or ischemia are found to have non‐obstructive coronary arteries (ANOCA/INOCA). Their symptoms can sometimes be explained by coronary microvascular dysfunction and/or vasospasm. Selecting patients for acetylcholine testing remains challenging, and the procedure is not trivial in terms of logistics, cost, and risk.

**Aims:**

The aim of this single‐center retrospective study was to identify predictors of coronary spasm and define features that may help avoid unnecessary testing.

**Methods:**

All consecutive ANOCA/INOCA patients who underwent intracoronary acetylcholine testing between February 2022 and June 2025 at a tertiary university hospital were included. Baseline clinical variables were analyzed using multivariable logistic regression. A 3‐variable exploratory model was then constructed to identify independent predictors of unnecessary acetylcholine testing.

**Results:**

Among the study population (*n* = 47), 46.8% demonstrated a positive spasm response. In the multivariable model, age ≥ 60 years (OR = 0.11, 95% CI [0.02–0.80]; *p* = 0.029) and dyslipidaemia (OR = 0.12, 95% CI [0.01–0.99]; *p* = 0.049) were independently associated with lower odds of spasm, whereas IMR ≥ 25 showed a non‐significant association with lower odds of spasm (OR = 0.10, 95% CI [0.005–1.9]; *p* = 0.128). The 3‐variable exploratory model showed good discrimination (AUC 0.83, 95% CI 0.68–0.97) and was associated with a reduction in overall ACh testing by 45.2% in complete‐case analysis, with a sensitivity of 85.7%.

**Conclusions:**

Dyslipidaemia and age ≥ 60 years are independently associated with lower odds of acetylcholine‐induced spasm. This model showed good discrimination and may help inform strategies to reduce unnecessary acetylcholine testing, although these findings require external validation.

**Abbreviations**
Ach: acetylcholine
ANOCA: angina with no obstructive coronary arteries
CFR: coronary flow reserve
CMD: coronary microvascular dysfunction
FFR: fractional flow reserve
IMR: index of microvascular resistance
INOCA: ischemia with no obstructive coronary arteries

## 1 Introduction

Angina or ischemia with non‐obstructive coronary arteries (ANOCA/INOCA) is a prevalent clinically relevant entity affecting up to 50% of patients undergoing coronary angiography [1, 2, 3]. Whilst previously considered benign, many patients with ANOCA/INOCA have underlying coronary vascular dysfunction—a term encompassing both coronary microvascular dysfunction (CMD) and coronary spasm. Crucially, both pathologies are linked to impaired quality of life, recurrent hospitalizations, and adverse cardiovascular outcomes [4, 5, 6, 7].

CMD, present in ~41% of ANOCA/INOCA patients, reflects impaired vasodilation and/or abnormal microvascular constriction [8]. It can be diagnosed invasively using thermodilution‐derived indices such as the index of microvascular resistance (IMR) and coronary flow reserve (CFR) [9, 10]. In contrast, abnormal vasoreactivity, manifesting as epicardial or microvascular spasm, requires pharmacologic provocation, most often with intracoronary acetylcholine (ACh) [11, 12].

The latest European guidelines highlight the importance of performing comprehensive invasive coronary function testing in patients with ANOCA/INOCA to diagnose underlying CMD, coronary vasospasm, or the coexistence of both conditions [13]. Despite these recommendations, ACh testing remains underutilized due to concerns regarding safety, procedural time, complex solution preparation, and operator experience.

Yet, when ACh testing is used systematically in patients with ANOCA/INOCA, only ~40%−60% ultimately have a positive test [14]. This suggests that testing may not be necessary in all patients with ANOCA/INOCA [15]. At present, no validated algorithm exists to identify patients most likely to benefit, leaving decisions to physician discretion. This contributes to inconsistent use, missed diagnoses, and unnecessary procedures in low‐probability cases [17, 18].

Prior studies have identified variables such as epicardial atherosclerosis, tortuosity, female sex, and endothelial dysfunction as predictors of vasospasm, but results have been inconsistent and rarely validated [19, 20, 21]. This study aimed to identify independent clinical predictors of epicardial or microvascular spasm, as defined by COVADIS criteria, in ANOCA/INOCA, to improve patient selection, diagnostic yield, and reduce unnecessary procedures.

## 2 Methods

### 2.1 Study Population and Procedures

This single‐center, retrospective cohort study was conducted at Lausanne University Hospital (CHUV), Switzerland, between February 2022 and June 2025. All consecutive patients recorded in the institutional coronary physiology registry who underwent intracoronary ACh provocation testing during this period were included. Patients in this registry had angina and/or objective evidence of myocardial ischemia with non‐obstructive CAD, defined as <50% luminal narrowing on angiography according to the 2024 ESC Guidelines for the management of chronic coronary syndromes. For lesions with intermediate (50%–70%) diameter stenosis, wire‐based fractional flow reserve (FFR) was measured and was negative in all cases (FFR > 0.80). Patients with missing baseline variables were retained for descriptive and univariate analyses, while complete‐case analysis was applied for the multivariable model. Baseline demographic, clinical, and angiographic data were obtained from the registry and electronic medical records.

### 2.2 Data Collection and Variables

A prespecified set of 12 clinical and angiographic variables was assessed as potential predictors of ACh‐induced coronary spasm: age, sex, hypertension, diabetes, dyslipidaemia, current smoking, family history of CAD, body mass index (BMI), angina type, CFR (bolus and continuous, analyzed separately), and IMR. Age was dichotomized at ≥ 60 years, and BMI at ≥ 30 kg/m^2^. Abnormal thresholds were IMR ≥ 25 and CFR < 2.5; in tables, we report CFR as > 2.5 (normal) for readability. The primary outcome was the occurrence of spasm, defined according to the Coronary Vasomotion Disorders International Study Group (COVADIS) criteria. Epicardial spasm was defined as a transient ≥ 90% luminal constriction accompanied by angina and ischemic ECG changes, whereas microvascular spasm was diagnosed when angina and ischemic ECG changes occurred in the absence of ≥ 90% epicardial narrowing.

### 2.3 Invasive Assessment

Coronary physiology was assessed via a pressure–temperature sensor–equipped guidewire (PressureWire X, Abbott Vascular, Santa Clara, CA, USA). CFR and IMR were measured by thermodilution. Incremental doses of intracoronary ACh were administered in the left coronary artery (2, 20, 100, and 200 µg cumulative), unless terminated earlier due to hemodynamic instability or the occurrence of spasm as defined by COVADIS criteria. In line with institutional practice, the protocol was restricted to the left coronary system. All procedural and physiological data were prospectively entered into a dedicated institutional registry and subsequently used for the present analysis. In patients undergoing comprehensive physiology assessment, coronary microcirculatory function was assessed prior to ACh testing, although CMD assessment was not performed systematically in all cases.

### 2.4 Statistical Analysis

The primary endpoint was ACh‐induced coronary spasm, defined as either epicardial or microvascular spasm according to COVADIS criteria. For modeling, predictors were analyzed as categorical variables, dichotomized for bedside interpretability, and are reported as frequencies and percentages. Baseline continuous variables (e.g., age) are summarized as mean ± SD and compared using Welch's *t*‐test (or Mann–Whitney when non‐normal); categorical variables with Fisher's exact (or *χ*² where appropriate).

All 12 prespecified variables were evaluated in a multivariable logistic regression analysis. Given the modest sample size, modeling was considered exploratory and aimed at identifying candidate variables associated with ACh‐induced spasm rather than developing a definitive prediction model. Variables meeting the significance threshold (*p* < 0.05), along with IMR ≥ 25 based on physiological relevance, were retained in the final 3‐variable exploratory model. In this dataset, age ≥ 60 years, dyslipidaemia, and IMR ≥ 25 were included. Model calibration was assessed with the Hosmer‐Lemeshow test, and discrimination with the area under the receiver operating characteristic (ROC) curve (AUC).

Because of the modest sample size and the potential risk of model optimism, internal validation was performed using bootstrap resampling (5000 iterations) to assess optimism in model performance. Bias‐corrected and accelerated (BCa) confidence intervals were calculated, and the bootstrap distribution of the area under the ROC curve was examined to assess the stability of model discrimination. Given the limited events‐per‐variable ratio, results should be interpreted cautiously and primarily as signal detection rather than stable effect estimation.

Clinical utility was examined by applying the optimal Youden index cut‐off to quantify overall ACh testing reduction and unnecessary tests avoided. A two‐sided *p* < 0.05 was considered statistically significant. Analyses were performed with SPSS version 29.0.2.0 (IBM Corp., Armonk, NY, USA) and R version 4.4.2 (R Foundation for Statistical Computing, Vienna, Austria).

### 2.5 Ethics Statement

The study was approved by the local institutional ethics committee, and all patients provided written informed consent at registry enrollment for use of their clinical data.

## 3 Results

Unless otherwise specified, all model‐based analyses refer to complete‐case data (*n* = 31).

### 3.1 Study Population and Baseline Characteristics

A total of 47 patients underwent intracoronary ACh testing, with 22 (46.8%) fulfilling the diagnostic criteria for coronary spasm. Of these, 19 (40.4% of the cohort; 86.4% of spasm‐positive) had epicardial spasm, and 3 (6.4% of the cohort; 13.6% of spasm‐positive) had microvascular spasm. Baseline demographic, clinical, and angiographic characteristics stratified by spasm status are presented in Table 1. Overall, the cohort was predominantly female with a mean age of 59.3 years and a high prevalence of conventional cardiovascular risk factors. A comprehensive CMD assessment was performed in 72.3% of patients with coronary thermodilution. In descriptive (unadjusted) comparisons, diabetes was less frequent in the spasm group (5% vs. 29%; *p* = 0.049). However, this association did not remain significant in multivariable analysis.

**Table 1:** Baseline characteristics of the study population.

Characteristics	Overall (*n* = 47)	Spasm + (*n* = 22)	Spasm‐ (*n* = 25)	*p* value^a^
Age ≥ 60 years	23/47 (49%)	8/22 (36%)	15/25 (60%)	0.147
Age, years (mean ± SD)	59.3 ± 12.1	58.6 ± 13.5	60 ± 10.9	0.700
Female sex	35/47 (74%)	17/22 (77%)	18/25 (72%)	0.747
Hypertension	23/46 (50%)	8/22 (36%)	15/24 (62%)	0.139
Diabetes	8/46 (17%)	1/22 (5%)	7/24 (29%)	**0.049**
Dyslipidaemia	27/46 (59%)	11/22 (50%)	16/24 (67%)	0.370
BMI ≥ 30 kg/m^2^	11/46 (24%)	4/21 (19%)	7/25 (28%)	0.514
Current smoker	10/47 (21%)	4/22 (18%)	6/25 (24%)	0.730
Family history of CAD	10/44 (23%)	6/22 (27%)	4/22 (18%)	0.721
Typical angina	34/47 (72%)	17/22 (77%)	17/25 (68%)	0.530
IMR ≥ 25 (abnormal, bolus thermodilution)	24/32 (75%)	13/14 (93%)	11/18 (61%)	0.053
CFR > 2.5 (normal, continuous thermodilution)	18/28 (64%)	8/11 (73%)	10/17 (59%)	0.689
CFR > 2.5 (normal, bolus thermodilution)	9/34 (26%)	4/15 (27%)	5/19 (26%)	1.000

^a^ Categorical variables are shown as *n*/*N* (%) and compared with Fisher's exact test; continuous variables as mean ± SD and compared with Welch's *t*‐test (two‐sided). Denominators reflect available data: hypertension (*n* = 46), diabetes (*n* = 46), dyslipidaemia (*n* = 46), BMI (*n* = 46), family history of CAD (*n* = 44), CFR by bolus thermodilution (*n* = 34), IMR by bolus thermodilution (*n* = 32), CFR by continuous thermodilution (*n* = 28). IMR is reported as ≥ 25 (abnormal) during adenosine hyperaemia; CFR > 2.5 denotes normal.

### 3.2 Identification of Predictors

**Figure 1:**
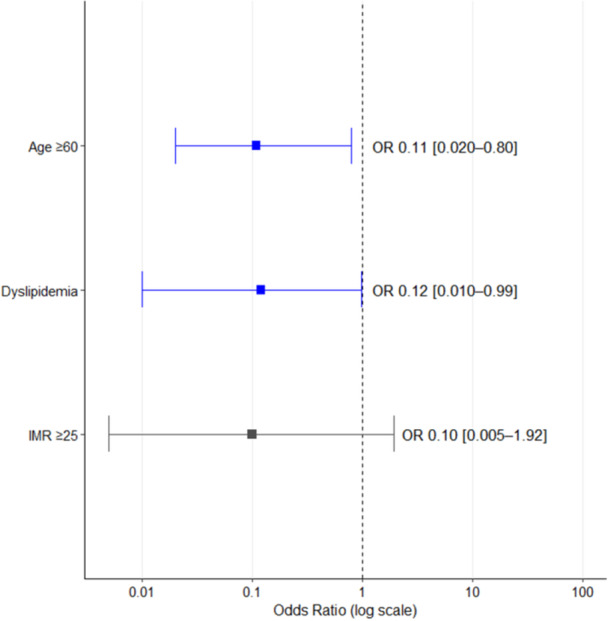
Predictors of ACh‐induced spasm in multivariable analysis. Forest plot displaying odds ratios (OR) with 95% confidence intervals (CI) for variables included in the multivariable logistic regression model. Age ≥ 60 years (OR 0.11, 95% CI [0.02–0.80]; *p* = 0.029) and dyslipidaemia (OR 0.12, 95% CI [0.01–0.99]; *p* = 0.049) were independently associated with lower odds of spasm, while IMR ≥ 25 showed a non‐significant association with lower odds of spasm (OR 0.10, 95% CI [0.005–1.9]; *p* = 0.128) (complete cases, *n* = 31). [Color figure can be viewed at wileyonlinelibrary.com]

After entering all 12 prespecified variables into a multivariable logistic regression model, two predictors met the significance threshold (*p* < 0.05), and IMR ≥ 25 was additionally retained based on physiological relevance in the final model: age ≥ 60 years, dyslipidaemia, and IMR ≥ 25. In this model, age ≥ 60 years (OR 0.11, 95% CI [0.02–0.80]; *p* = 0.029) and dyslipidaemia (OR 0.12, 95% CI [0.01–0.99]; *p* = 0.049) were independently associated with lower odds of spasm, whereas IMR ≥ 25 showed a non‐significant association with lower odds of spasm (OR 0.10, 95% CI [0.005–1.9]; *p* = 0.128). Results are illustrated in Figure 1.

### 3.3 Model Performance

**Figure 2:**
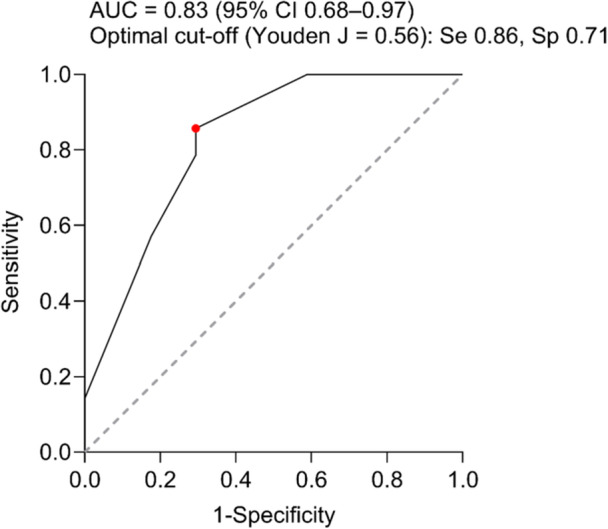
ROC curve of the 3‐variable exploratory model for acetylcholine‐induced spasm. Receiver operating characteristic (ROC) curve for the 3‐variable exploratory model. The model showed good discrimination with AUC 0.83 (95% CI 0.68–0.97). The optimal cut‐off by the Youden index (*J* = 0.56) is marked in red, yielding sensitivity 85.7% and specificity 70.6% (complete cases, *n* = 31). [Color figure can be viewed at wileyonlinelibrary.com]

The 3‐variable exploratory model achieved an AUC of 0.83 (95% CI [0.68–0.97]), indicating good discrimination (Figure 2). Calibration was acceptable (Hosmer‐Lemeshow *p* = 0.913), with Nagelkerke *R*² = 0.48 (Table 2). At the Youden‐optimal cut‐off, the model achieved a sensitivity 85.7%, a specificity 70.6%, a positive predictive value 70.6%, a negative predictive value 85.7%, and an overall accuracy 77.4%. Internal validation using bootstrap resampling confirmed the stability of the exploratory model, with the bootstrap distribution of the AUC centered around the apparent AUC of 0.83 (95% BCa CI 0.67–0.95).

**Table 2:** Performance of the 3‐variable exploratory logistic regression model (age ≥ 60 years, dyslipidaemia, IMR ≥ 25).

Metric	Value
AUC (95% CI)	0.83 (95% CI 0.68–0.97)
Hosmer‐Lemeshow *p*	0.913
Nagelkerke *R* ^2^	0.48
Classification accuracy	77.4% (24/31)
Sensitivity at Youden cut‐off	85.7% (12/14)
Specificity at Youden cut‐off	70.6% (12/17)
PPV at Youden cut‐off	70.6% (12/17)
NPV at Youden cut‐off	85.7% (12/14)

Abbreviations: AUC = area under the ROC curve; *p* from the Hosmer–Lemeshow test; *R*² = Nagelkerke coefficient of determination.

*Note:* Values from complete‐case analysis (*n* = 31). Confusion matrix at the Youden‐optimal cut‐off: TP 12, FP 5, TN 12, FN 2.

### 3.4 Clinical Utility Analysis

**Figure 3:**
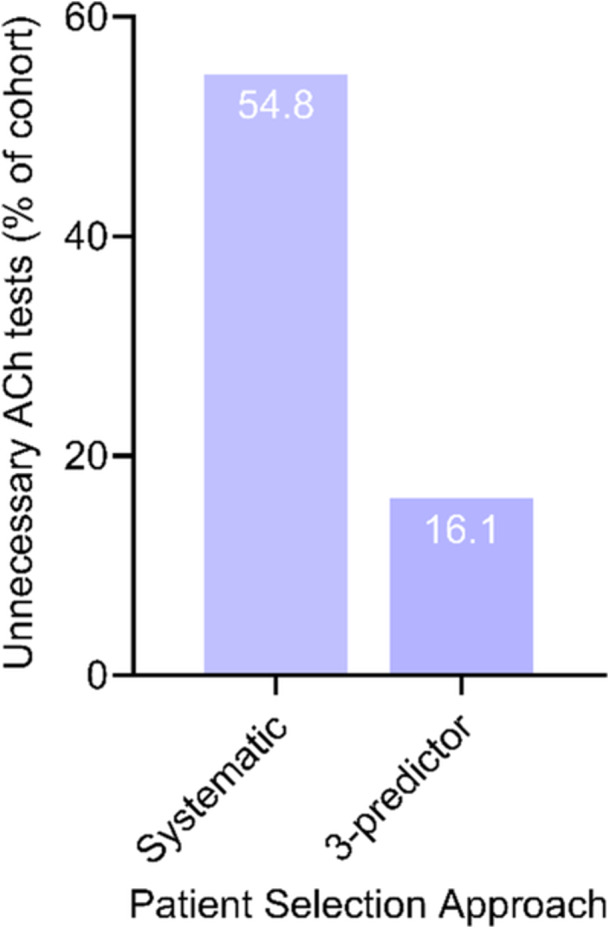
Unnecessary acetylcholine tests under a systematic versus 3‐variable exploratory model–guided strategy. Unnecessary ACh tests decreased from 54.8% (17/31) to 16.1% (5/31), corresponding to a reduction of 12/17 unnecessary tests (70.6%) (complete cases, *n* = 31). [Color figure can be viewed at wileyonlinelibrary.com]

**Figure 4:**
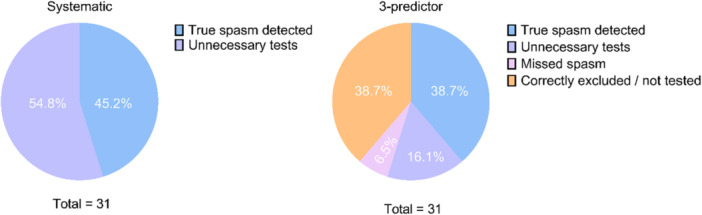
Comparison of systematic versus 3‐variable exploratory model–guided strategies for ACh testing. With systematic testing, 54.8% (17/31) of procedures were unnecessary, 45.2% (14/31) were true spasms, and no spasms were missed. With the 3‐variable exploratory model, unnecessary tests fell to 16.1% (5/31); correctly excluded/not tested were 38.7% (12/31); missed spasm 6.5% (2/31); true spasm detected 38.7% (12/31). Overall, this corresponds to a reduction of 12/17 unnecessary tests (70.6%), and an absolute reduction of 12/31 (38.7%) (complete cases, *n* = 31). [Color figure can be viewed at wileyonlinelibrary.com]

The overall comparison between systematic ACh testing and the 3‐variable exploratory model–guided strategy is shown in Figure 3. Under systematic testing, 54.8% of procedures were unnecessary, and 45.2% detected true spasm. With the 3‐variable exploratory model, unnecessary tests fell to 16.1%, true spasm detected was 38.7%, and 38.7% were correctly spared. This corresponded to a 70.6% reduction in unnecessary tests and a 45.2% reduction in overall testing, at the cost of 2 missed spasms (6.5%) (Figure 4).

## 4 Discussion

In this single‐center ANOCA/INOCA cohort, we identified simple clinical and physiological predictors that stratified the likelihood of vasoreactivity on ACh testing. Age ≥ 60 years and dyslipidaemia were independently associated with lower odds of spasm, and IMR ≥ 25 showed a non‐significant association with lower odds of spasm. A model combining these factors achieved good discrimination (AUC 0.83), suggesting that a substantial proportion of unnecessary ACh tests could potentially be avoided. However, this came at the cost of two missed epicardial spasms, underscoring the trade‐off between efficiency and sensitivity.

At the bedside, these three predictors may help guide decisions on whether to pursue ACh testing in patients with suspected ANOCA/INOCA. The clinical interpretation is intuitive: older patients and those with dyslipidaemia are more likely to have alternative explanations for angina, such as underlying microvascular dysfunction (CMD), rather than vasospastic disease. Contemporary literature highlights the substantial overlap between vasospastic angina and CMD and emphasizes the need for integrated interpretation of invasive coronary function testing rather than strict separation of these entities [22]. This is consistent with our observation that higher IMR values (≥ 25) were associated with lower odds of spasm; although this association was not statistically significant and confidence intervals were wide, the direction of effect is consistent with the hypothesis that vasospasm may be more likely when baseline microvascular resistance is lower. The apparent discrepancy between univariate and multivariable associations may reflect statistical instability related to the limited sample size. Taken together, these findings reinforce the concept that integrating simple clinical features with physiology can improve patient selection for invasive vasoreactivity testing.

### 4.1 Comparison With Previous Studies

Several clinical factors have been proposed as predictors of increased vasospasm risk, including female sex, smoking, and coronary tortuosity, but results across studies have been heterogeneous and often not consistently replicated [19, 20, 21]. Although diabetes appeared less frequent among spasm patients in descriptive (unadjusted) comparisons, this association did not persist after adjustment, consistent with prior reports of heterogeneous findings. In our study, two readily available risk factors, older age and dyslipidaemia, were instead associated with *lower* odds of spasm. This observation contrasts with prior literature, which has generally emphasized positive rather than protective associations, and suggests that in patients with a heavier burden of traditional risk factors, alternative mechanisms such as CMD may predominate.

Recent evidence has highlighted that differences in diagnostic criteria used during intracoronary ACh provocation testing may influence both the classification of vasospastic phenotypes and associated clinical outcomes, further supporting the need for careful interpretation across studies [23]. Given the limited sample size, statistical variability cannot be excluded, but these findings suggest that both demographic and physiological markers may be integrated to refine patient selection for vasoreactivity testing.

### 4.2 Clinical Implications

Despite guideline recommendations, ACh testing remains underutilized in many centers, partly due to concerns about safety, additional procedure time, the practical demands of ACh solution preparation, and uncertain incremental yield [16, 17]. Tools that help identify patients more or less likely to demonstrate spasm could improve efficiency by focusing pharmacologic testing where diagnostic yield is highest. Although the exploratory model suggested that some tests might potentially be avoided, this must be balanced against the risk of missed vasospasm. Accordingly, the present findings should not be interpreted as supporting the use of the model as a clinical decision tool. Although two epicardial spasms were missed, the overall trade‐off between efficiency and sensitivity was modest, suggesting that structured prediction tools may help inform test allocation. The model is not intended for clinical decision‐making but to inform hypothesis generation and future prospective validation.

### 4.3 Methodological Considerations and Limitations

Several limitations merit consideration. First, this was a retrospective, single‐center study, which limits generalizability and may introduce selection bias. The modest sample reduced statistical power, particularly for less common predictors such as diabetes, and may have resulted in failure to detect true associations. Given the modest sample size and data‐driven variable retention, the present model should be considered exploratory and hypothesis‐generating rather than a definitive clinical prediction tool. Additionally, predictor retention based on statistical significance may introduce selection bias and inflate apparent model performance, further supporting the exploratory nature of the present analysis. Second, the definition of outcome relied on the COVADIS criteria, which represent the current consensus standard but remain partly operator dependent. ACh testing was performed only in the left coronary artery, in accordance with institutional practice. This approach may underestimate the prevalence of coronary spasm, particularly in patients with right coronary artery involvement or right‐dominant coronary circulation. Third, not all patients underwent complete physiology assessment (CFR and IMR were available only in subsets), reducing statistical power for these variables and potentially biasing results toward clinical predictors.

Finally, our analysis focused solely on predictors of spasm occurrence and did not assess long‐term outcomes. Whether these predictors also stratify risk for recurrent angina, hospitalizations, or adverse cardiovascular events cannot be determined from this dataset. Future prospective multicentre studies integrating clinical predictors, invasive physiology, and patient‐centered outcomes are needed to confirm these findings and establish clinical utility.

## 5 Conclusion

In this single‐center cohort of ANOCA/INOCA patients undergoing systematic ACh testing, age ≥ 60 years and dyslipidaemia were independently associated with lower odds of ACh‐induced spasm, while IMR ≥ 25 showed a non‐significant association with lower odds. A simple 3‐variable exploratory model demonstrated good discrimination, although two epicardial spasms would have been missed using the exploratory model‐based strategy. Given the modest sample size and exploratory modeling approach, these findings should be interpreted as hypothesis‐generating and require external validation in larger multicentre cohorts before any clinical application.

## Impact on Daily Practice

## What Is New?

- In ANOCA/INOCA patients, age ≥ 60 years and dyslipidaemia were independently associated with lower odds of acetylcholine‐induced spasm.
- A 3‐variable exploratory model (age ≥ 60 years, dyslipidaemia, IMR ≥ 25) showed good discrimination (AUC 0.83).
- Applying this model may reduce overall ACh testing and unnecessary testing, although this requires external validation.

## What Are the Clinical Implications?

- Simple clinical factors may guide selection for vasoreactivity testing and improve diagnostic efficiency.
- Structured prediction tools could reduce procedural burden and resource use, but require external validation.
- Integrating demographic and physiological markers may enable more personalized invasive testing strategies.

## Conflicts of Interest

Dr Stéphane Fournier has received honoraria or research support from Abbott, Edwards Lifesciences, CathWorks, Medtronic, Amgen, and Bayer. The other authors declare no conflicts of interest.

## Data Availability

The data that support the findings of this study are available on request from the corresponding author. The data are not publicly available due to privacy or ethical restrictions.
